# Anti-Synthetase Syndrome-Related Interstitial Lung Disease With Anti-PL-12 Antibodies

**DOI:** 10.7759/cureus.12936

**Published:** 2021-01-27

**Authors:** Belqis Elferjani, Adnan Liaqat, Mohammed Zaman, Marvin Sexton

**Affiliations:** 1 Internal Medicine, Southeast Health Medical Center, Dothan, USA; 2 Pulmonology, Dothan Pulmonary Associates, Dothan, USA

**Keywords:** anti-synthetase syndrome, interstitial lung disease, anti-pl-12 antibody

## Abstract

Anti-synthetase syndrome usually comprises interstitial lung disease, myositis, arthralgias, and Raynaud phenomenon. The anti-PL-12 antibody is directed against the enzyme alanyl-tRNA synthetase and has been associated with interstitial lung disease in the absence of inflammatory myositis. We report the case of a 33-year-old woman with complaints of progressive dyspnea, a persistent dry cough, along with intermittent low-grade fever for a few months. A computed tomography (CT) scan of the chest showed the presence of patchy bilateral airspace opacities and infiltrates. It also showed significant mediastinal and hilar lymphadenopathy. Bronchoscopy with transbronchial biopsy was performed, and histopathology changes were consistent with connective tissue disease related to interstitial lung disease. Further workup revealed the presence of anti-PL-12 antibodies. This case illustrates a rare association of interstitial lung disease with the anti-PL-12 antibody.

## Introduction

Anti-synthetase syndrome (AS) is a rare and chronic autoimmune disease with autoantibodies directed against amino-acyl transfer RNA synthetase. Its clinical features range from arthritis, myositis, Raynaud’s phenomenon, mechanic’s hands, to interstitial lung disease [1]. The interstitial lung disease in anti-synthetase syndrome carries a poor prognosis with increased morbidity and mortality when compared with other inflammatory myopathies [2].

Aminoacyl-tRNA synthetase autoantibodies were first identified in the 1980s [3]. In the 1990s, it became well-known that these antibodies are linked to distinct clinical features, leading to the official recognition of anti-synthetase syndrome [4-5]. Anti-Jo 1 antibodies are most common (20%), whereas anti-PL-12 antibodies (alanyl-tRNA synthetase) occur in <3% of known cases.

Anti-synthetase syndrome usually comprises interstitial lung disease, myositis, arthralgia, and Raynaud phenomenon. The anti-PL-12 antibody is directed against the enzyme alanyl-tRNA synthetase and has been associated with interstitial lung disease in the absence of inflammatory myositis. This case illustrates a rare association of interstitial lung disease with the anti-PL-12 antibody.

## Case presentation

We report the case of a 33-year-old woman with a past medical history significant for asthma, iron deficiency anemia, and former smoking who presented with complaints of progressive dyspnea, persistent dry cough, and two recent episodes of pneumonia that was adequately treated. She further had a 10-month history of intermittent, low-grade fever associated with gradual weight loss over the same time period. She denied any hemoptysis, chills, or occupational dust exposure. She had no recent administration of medications known to cause anatomic or pathological pulmonary abnormalities.

**Figure 1:**
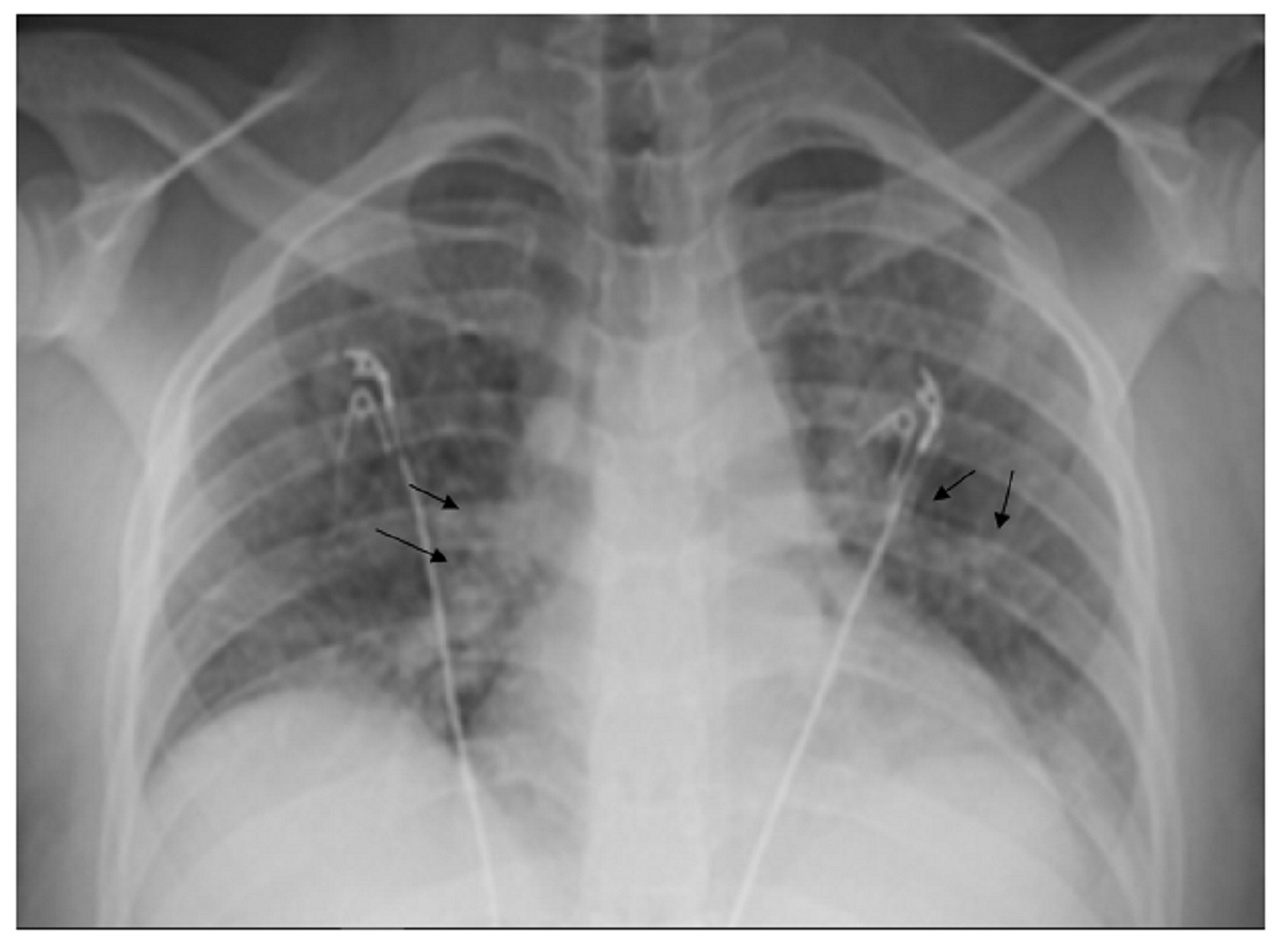
Chest X-ray showing bilateral interstitial and airspace opacities

Initial lab work included white blood cell (WBC) 12.3 × 10^9^/L, Hg 9.1 mg/dl with mean corpuscular volume (MCV) 61.5, platelets 565× 10^9^/L, creatinine 0.54 mg/dl, Na 138 mEq/L, K 4.4 mEq/L, aspartate aminotransferase (AST) 12 units/L, alanine transaminase (ALT) 4 units/L. Lactate was 0.56 mmol/L and D-dimer was 219 ng/mL. Urinalysis was negative for any significant findings. Partial thromboplastin time (PTT) was 29.4 seconds, prothrombin time (PT) 13.3 seconds, and international normalized ratio (INR) 1.17. The initial chest X-ray is shown in Figure 1.

**Figure 2:**
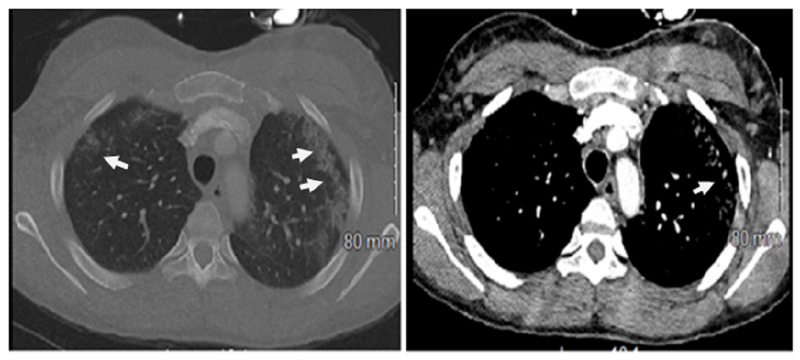
Computed tomography of the chest showing bilateral interstitial opacities, ground-glass opacities, and bronchiectasis

The computed tomography (CT) scan of the chest showed the presence of bilateral interstitial opacities, ground-glass opacities, subpleural nodules, and bronchiectasis. It also showed significant mediastinal and hilar lymphadenopathy. CT images are shown in Figure 2.

Both the viral respiratory infectious panel and hypersensitivity pneumonitis panel were negative. The influenza screen was negative. The hepatitis B surface antigen (HBsAg) and hepatitis B surface antibody (HBsAb) were negative. The C-3 and C-4 complement levels were within the normal range, 171 mg/dl and 42.7 mg/dL, respectively. The Quantiferon TB (tuberculosis) gold test was negative. Serum aldolase was 6.9 U/L and total creatinine kinase (CK) was 81 U/L. 

**Figure 3:**
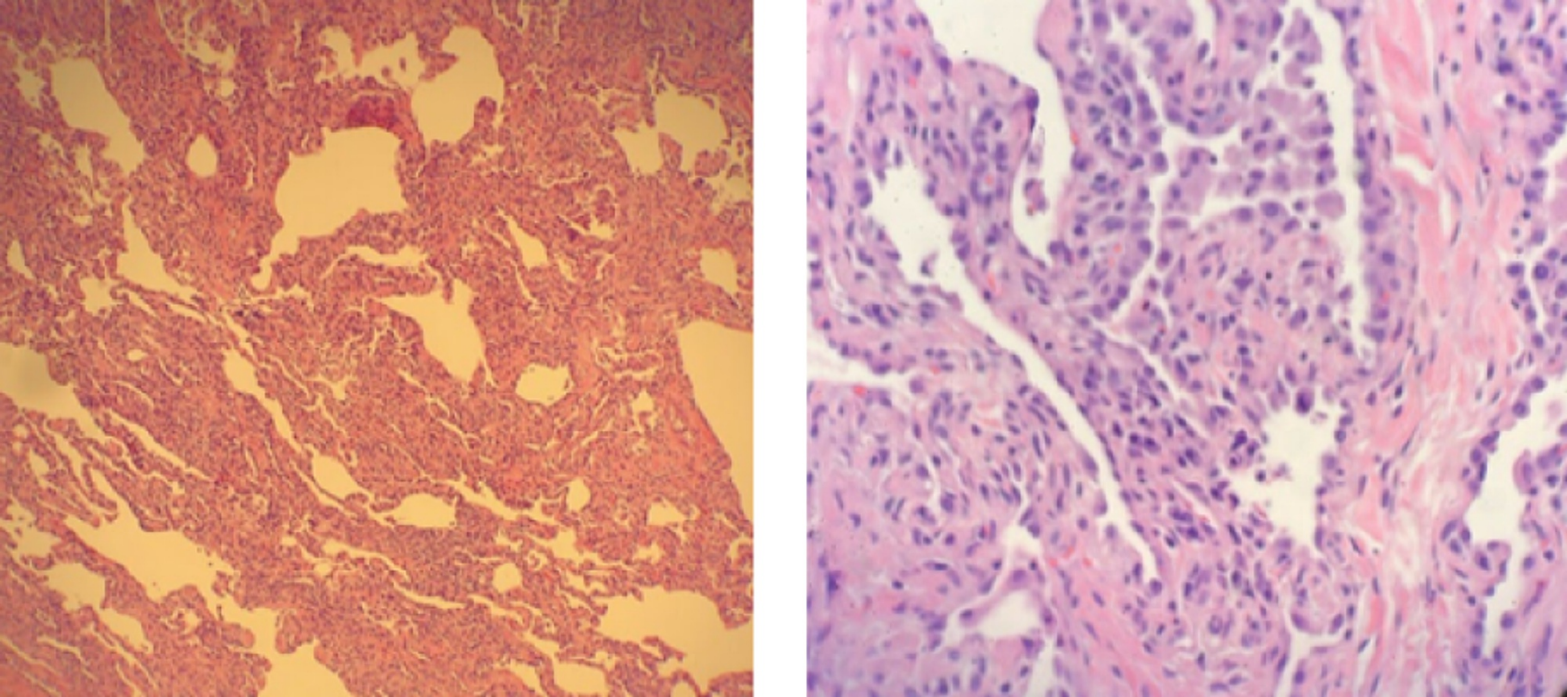
Histopathology from transbronchial biopsy sample showing a pattern of fibrosis obscuring the normal alveolar architecture consistent with chronic cellular and fibrosing interstitial pneumonia

Bronchoscopy with bronchoalveolar lavage (BAL) and transbronchial biopsy was performed, and histopathology changes were consistent with connective tissue disease related to interstitial lung disease as shown in Figure 3.

The BAL culture was negative for any organism, including acid-fast bacilli (AFB). The serology panel for all antibodies was negative except for PL-12 autoantibodies as shown in Table 1.

**Table 1:** Illustrates positivity for the anti-PL-12 antibody ANA: antinuclear antibodies

Serology	Results	Serology	Results
ANA	1:1280	Anti-SCL-70	Negative
Aldolase	Negative	Anti-SM	Negative
Anti-ds-DNA	Negative	Anti-SSA	Negative
Anti-SSB	Negative	Anti-U1RNP	Negative
Anti-Jo-1	Negative	Anti-PL-7	Negative
Anti-EJ	Negative	Anti-PL-12	Positive

## Discussion

Inflammatory myopathies include a heterogeneous group of autoimmune disorders with various clinical subgroups. AS is one of the major subgroups with anti-synthetase antibodies. AS is characterized by varying degrees of interstitial lung disease (ILD), myositis, arthropathy, fever, Raynaud's phenomenon, and mechanic's hands, and the morbidity and mortality of the disease are usually linked to pulmonary findings. Corticosteroids are the mainstay of acute therapy, although treatment often requires immunosuppressant medications such as cyclophosphamide, azathioprine, mycophenolate mofetil, cyclosporine, tacrolimus, or rituximab [6]. AS syndrome with ILD carries a grim prognosis and the presence of AS antibodies is the strongest predictor of ILD development [7].

Our patient with AS syndrome-related ILD and positive anti-PL 12 antibodies had no associated myositis. To our knowledge, the absence of myositis in AS syndrome with anti-PL 12 antibodies is relatively rare and there are only a few case reports reported so far. After a detailed review of such cases, we compiled a table of such cases (Table 2).

**Table 2:** Literature review of the reported cases on the anti-PL-12 antibody-associated anti-synthetase syndrome with clinical presentation and management ILD: interstitial lung disease; CyA: cyclosporine A; mPSL: methylprednisolone; IVCY: intravenous cyclophosphamide; RTX: rituximab; IVIG: intravenous immunoglobulin

	Author	Reference	Age/sex	Presentation	Diagnosis	Myositis	Treatment	Outcomes
Case 1	Ghysen K. et al.	[8]	64, M	Dry cough; progressive dyspnea; arthralgia	ILD related to the anti-synthetase syndrome	No	Steroid and azathioprine	Good response
Case 2	Tokunaga K, H. et al.	[9]	65, F	Fever and dry cough, Dyspnea	ILD	Yes	mPSL; CyA; IVCY; with concomitant RTX cycles	Improving
Case 3	Yahaba M, S. et al.	[10]	37, F	Extreme proximal muscle weakness	Necrotizing autoimmune myositis in the association of ILD	Yes	Steroid; IVIG	Improving
Case 4	Satoh, H. et al.	[11]	55, F	Progressive dyspnea and polyarthralgia	Interstitial pneumonia	No	Steroid	Improving

Interstitial disease can be the only manifestation of the anti-synthetase syndrome in the absence of inflammatory myositis. This case highlights the significance of considering anti-synthetase antibodies in patients with interstitial lung disease with no other signs of connective tissue disease.

## Conclusions

We conclude that the anti-PL-12 antibody is associated with the presence of ILD, but it differs from other antibodies associated with the anti-synthetase syndrome, like anti-jo-1 and anti-7, due to the absence of myositis and arthritis. The anti-PL-12 antibody can be an underlying cause of idiopathic ILD. Finally, further investigation is required to understand this disease entity and to improve diagnostic and therapeutic strategies.
